# Estimating the burden of rhodesiense sleeping sickness during an outbreak in Serere, eastern Uganda

**DOI:** 10.1186/1471-2458-8-96

**Published:** 2008-03-26

**Authors:** Eric M Fèvre, Martin Odiit, Paul G Coleman, Mark EJ Woolhouse, Susan C Welburn

**Affiliations:** 1 Centre for Infectious Diseases, University of Edinburgh, Ashworth Laboratories, West Mains Road, Edinburgh, EH9 3JT, UK; 2 Centre for Tropical Veterinary Medicine, College of Medicine and Veterinary Medicine, University of Edinburgh, Easter Bush, Roslin, Midlothian, EH25 9RG, UK; 3 Uganda AIDS Control Project, P.O. Box 25589, Kampala, Uganda; formerly Sleeping Sickness Programme, Livestock Health Research Institute, P. O. Box 96 Tororo, Uganda; 4 London School of Hygiene and Tropical Medicine, University of London, Keppel Street, WC1 7HT, UK

## Abstract

**Background:**

Zoonotic sleeping sickness, or HAT (Human African Trypanosomiasis), caused by infection with *Trypanosoma brucei rhodesiense*, is an under-reported and neglected tropical disease. Previous assessments of the disease burden expressed as Disability-Adjusted Life Years (DALYs) for this infection have not distinguished *T.b. rhodesiense *from infection with the related, but clinically distinct *Trypanosoma brucei gambiense *form. *T.b. rhodesiense *occurs focally, and it is important to assess the burden at the scale at which resource-allocation decisions are made.

**Methods:**

The burden of *T.b. rhodesiense *was estimated during an outbreak of HAT in Serere, Uganda. We identified the unique characteristics affecting the burden of rhodesiense HAT such as age, severity, level of under-reporting and duration of hospitalisation, and use field data and empirical estimates of these to model the burden imposed by this and other important diseases in this study population. While we modelled DALYs using standard methods, we also modelled uncertainty of our parameter estimates through a simulation approach. We distinguish between early and late stage HAT morbidity, and used disability weightings appropriate for the *T.b. rhodesiense *form of HAT. We also use a model of under-reporting of HAT to estimate the contribution of un-reported mortality to the overall disease burden in this community, and estimate the cost-effectiveness of hospital-based HAT control.

**Results:**

Under-reporting accounts for 93% of the DALY estimate of rhodesiense HAT. The ratio of reported malaria cases to reported HAT cases in the same health unit was 133:1, however, the ratio of DALYs was 3:1. The age productive function curve had a close correspondence with the HAT case distribution, and HAT cases occupied more patient admission time in Serere during 1999 than all other infectious diseases other than malaria. The DALY estimate for HAT in Serere shows that the burden is much greater than might be expected from its relative incidence. Hospital based control in this setting appears to be highly cost-effective, highlighting the value of increasing coverage of therapy and reducing under-reporting.

**Conclusion:**

We show the utility of calculating DALYs for neglected diseases at the local decision making level, and emphasise the importance of improved reporting systems for acquiring a better understanding of the burden of neglected zoonotic diseases.

## Background

Human African trypanosomosis (HAT), also known as sleeping sickness, is caused by *Trypanosoma brucei gambiense *or *T. b. rhodesiense*, the former occurring in West and Central Africa and the latter restricted to East Africa, with a predominant focus in eastern Uganda. HAT is a fatal disease if not treated [1], and during the clinical disease, patients suffer a variety of debilitating symptoms and sequelae. A commonly used index for expressing the burden of disease is disability adjusted life years (DALYs), a generic health measure incorporating both mortality and morbidity and used to gauge the relative public health importance of different diseases [2,3]. WHO/World Bank burden of disease studies [4] estimate the total DALY for HAT to be 1.53 million. For Africa, this compares to 40.9 million for malaria, 9.27 million for tuberculosis, 1.33 million for schistosomiasis and 2.01 million for lymphatic filariasis. Amongst the human infectious and parasitic diseases in Africa, human trypanosomiasis is ranked ninth and thirteenth out of 25 for mortality and DALYs, respectively, while among vector-borne diseases, HAT ranks second and fourth, respectively. Given that HAT tends to occur where there is a breakdown in control and that it affects mainly rural poor people, it is under-reported compared to other diseases [5,6]; this situation is mirrored with other neglected diseases [7,8]. Unlike malaria, HAT is always fatal if not treated and the costs of treatment, usually requiring hospitalisation, are high [9]. Because of this, it is particularly important to assess the cost burden to the health services in addition to the aggregate health burden to individual patients [10,11].

*T. b rhodesiense *HAT is an acute zoonotic disease and the available evidence supports the hypothesis that cattle are an important reservoir [12-14]. While often considered together, rhodesiense and gambiense HAT are different diseases both clinically and epidemiologically [15]; because rhodesiense HAT is less prevalent and less widely distributed than gambiense HAT, current published estimates of HAT morbidity are based on parameters of the latter [16,17] – eg using a disability weighting of 0.35 for each non-fatal case [18]. However, in its major focus of eastern Uganda, it has been responsible for many deaths throughout the 20^th ^century [19], and because of its regional importance in eastern Africa, focal pattern of occurrence, rapid progression, and zoonotic nature [20], it is important to specifically estimate the burden of rhodesiense HAT in communities. Importantly, trypanosomiasis control in this area could have both public health and veterinary benefits.

This paper examines how the unique features of rhodesiense HAT influence its burden on the local population and health services in Serere, part of Soroti District, eastern Uganda. This is the focal point for patients reporting during an ongoing epidemic of rhodesiense HAT [21]. In addition, these estimates are compared to local burden of disease estimates for malaria and other conditions in the same health unit over the same time period. These estimates are important for understanding the burden of rhodesiense HAT generally, and for assisting the decision-making process at sub-national or district levels in affected countries – an increasingly important objective [22-27]. Recently, DALYs have been calculated at the village level for *T.b. gambiense *in the Democratic Republic of Congo [17].

## Methods

### Health burden indices

We estimate two health burden indices for a rhodesiense HAT outbreak in Serere health sub-district, in north-eastern Uganda; firstly the DALY, and secondly an estimate of the costs to the health sub-district based on the total costs of hospitalization and treatment for the disease. Both measures of burden were estimated for one calendar year, using the variability in the data from 1999–2005 to capture the uncertainty in our estimates.

### Disability Adjusted Life Years (DALYs)

DALY calculations followed the general methods used in the first Global Burden of Disease study [2,16], and subsequent World Health Report estimates [4], employed a discount rate of 3% [2] and were conducted with and without age-weighting [28]. The features of rhodesiense HAT that need to be considered in DALY estimation are the age and sex profile of patients, the severity of illness, the case fatality rate and the duration of morbidity. The information required to calculate the DALY is summarized in Table 1. Our calculations follow a stochastic framework; the age-specific YLL, YLD and DALY are estimated using a stochastic model that captures both uncertainty (from estimates) and annual variability in the input data (data inputs are drawn from the years 1999–2005), and reflects this uncertainty and variability in the model outputs. We run 10,000 Monte-Carlo simulations of the model employing Latin Hypercube sampling [29], using the @Risk software package (Palisade, Newfield, NY, USA; version 4.05); our model is constructed to consistently sample values for input variables from the data from the same year at each iteration. We report outputs with their 95% confidence intervals.

**Table 1:** Information required for the estimation of disability-adjusted life years (DALYs) for rhodesiense sleeping sickness

Information type	Specific data required	Data sources
Years-of-life-lost (YLL)	1) Number of deaths	- health unit records of case fatality by age/sex
		- case under-reporting estimates (by age and sex, based on Odiit *et al*. [30] and Fèvre *et al*. [21])
	2) Life expectancy	- Uganda-specific life table for 2000 [31]
	3) Distribution of age at death	- health unit records of case age (with or without age-weighted case fatality rate and under-reporting rate)
Years-of-life-lived with disability (YLD)	1) Disability weighting	- expert opinion – see text (rhodesiense disability weighting not previously explicitly provided [16, 35])
	2) Duration of illness	- pre-admission from Odiit *et al*. [37]
		- post-admission from health unit records of cases
	3) Age-weighting of productivity	- health unit records of case age

### Years of life lost (YLL)

The number of HAT deaths in Serere health sub-district, was estimated as a function of both the number of reported deaths and the rate of case under-reporting. Our estimate of the rate of under-reporting is based on a previously published methodology [30] in which the early:late stage ratio is a crucial parameter. Two under-reporting rates, 0% and 69% were applied, representing complete reporting for the former and a previous estimate for this population [21] for the latter. This under-reporting rate was applied to model the number of unreported cases each year, by multiplying the rate by the total number of male and female cases presenting in each age group from 1999–2005. Thus, for every ten cases presenting at the health facility, an additional seven (95% CI 4–10.2) were estimated to be unreported in the community. All unreported cases are assumed to remain undetected, receiving no treatment and resulting in death. To reflect the uncertainty in the rate of under-reporting, the value of this parameter was, at each iteration of the model, drawn from a gamma distribution with parameters α = 20.063 and β = 0.034. The life expectancy at the age of death was based on a Uganda-specific life-table with a life expectancy at birth of 45 years [31]. Using national (rather than the a model – eg United Nations West 26) life table for regional comparisons is consistent with established methodologies [16,32,33]. The distribution of age of death was estimated from the distribution of ages of all cases of HAT admitted to Serere health centre during 1999–2005, though all inputs to the calculations were drawn, on each iteration of the model, from the same calendar year. We assumed that the case fatality rate is constant across age; while there are no *T.b. rhodesiense*-specific studies on this, recent work on *T.b. gambiense *has shown this to be a reasonable assumption [34].

### Years of life lived with Disability (YLD)

The disability weighting used for HAT up to the present is 0.35 [16,35] based on clinical cases of *T. b. gambiense. T.b. rhodesiense *HAT is a more acute disease, with a greater degree of incapacity suffered throughout the period of infection than *T.b. gambiense*; untreated cases die within 3–9 months of infection, vs. up to 2–3 years for *T.b. gambiense *[9,19]. Additionally, HAT occurs in two stages: stage 1 occurs while the parasite is present in the blood and lymphatic system, and stage 2 when the parasite has crossed the blood-barrier and infects the central nervous system. The previously used disability weighting for HAT [16] does not account for the sequelae associated with the different stages. Here, we apply different disability weights for early and late stage disease, and use weights appropriate for rhodesiense HAT. Murray and Lopez [2] describe a range of disability weight classifications that are determined by the severity of disability – based on the clinical expert opinion of one of the authors (MO) over 15 years of *T.b. rhodesiense *HAT treatment, we have used a disability weighting of 0.21 (equivalent to that for malaria) for early stage *T.b. rhodesiense *HAT and 0.81 for late stage *T.b. rhodesiense *HAT (though we also present a final output using 0.35 for late stage HAT for comparative purposes, to represent uncertainty about the disability weight [36]). A weight of 0.81 equates to the inability to undertake basic activities associated with daily living without assistance. In our analysis, early stage disability was applied to both early and late stage patients (assuming that patients presenting in the late stage had previously suffered early stage symptoms), and late stage disability was applied to late stage patients alone, in each case for the estimated duration of the symptoms associated with each stage. Duration of illness pre-admission in each stage were taken from Odiit *et al*. [37]; median time of illness pre-admission for early and late stage were 21 days and 61 days, respectively. The median duration of hospital stay for non-fatal cases in either the late or early stage (38 days) was obtained from the HAT case records at Serere Health Centre for 1999 (the data quality for this parameter for other years was not sufficient to include them). We used a non-parametric bootstrap to model the uncertainty in the duration of early and late stage illness, as well as the duration of hospital stay. Briefly, the non-parametric bootstrap estimates the uncertainty in a parameter by re-sampling from the distribution of the sample itself. The advantage of this method is that it allows us to model uncertainty in the time variables of the model without making any distributional assumptions about the data. The duration of illness estimate did not account for post-treatment disability; physical and mental retardation in children caused by HAT has been reported [38], as have other longer term sequelae, but they are not considered here. Our estimates should therefore be considered conservative.

### Costs to the local health system

The costs to the health system per HAT patient were estimated as the product of the total annual hospital stay in days and a standard daily cost. Total hospital stay was determined by multiplying the mean stay per patient (using a non-parametric bootstrap, as above, based on all patients diagnosed parasitologically in Serere during 1999) by the total number of patients in any given year (1999–2005), over 10,000 model iterations. A standard cost range per hospital day in 1999/2000 was estimated in consultation with staff at the Serere health centre and applied to all years. They considered that daily hospital costs were similar for all diseases for which patients were admitted as inpatients to their health centre and estimated it at between 3000–4000 Uganda Shillings (US$ 1.75 to US$ 2.35 per patient, with a most likely value of US$ 2 per patient). We used a betapert distribution to model this parameter. The drug costs for a full course of HAT treatment are estimated to be US$ 35 and US$ 63 for early and late stage patients respectively (WHO, 1998); these were modelled as point estimates. This does not include patient costs such as travel to hospital, which have been examined elsewhere for both *T.b. rhodesiense *[39] and *T.b. gambiense *[17]. Using these data we have also modelled the cost-effectiveness of the hospital-based intervention at preventing a loss of DALYs due to HAT, expressed as US$/DALYs averted.

### Relative burden of HAT compared to other diseases

The most commonly diagnosed illness in the study area was malaria. The DALY for malaria was estimated using data from all diagnosed (either clinically or parasitologically) inpatient and outpatient malaria cases and recorded deaths at the Serere health centre during 1999; non-HAT data were only available for 1999. For the YLL calculation, each reported death due to malaria in 1999 and its age at death were obtained. For the YLD calculation, a disability weighting of 0.21 per episode of all types of malaria was used [16] for all age groups; duration of morbidity for malaria was 0.01 years for an outpatient case irrespective of age, and 0.03 and 0.07 years for inpatients <5 and ≥ 5 years old, respectively [16].

We also compared the relative impact of HAT, malaria and tuberculosis in different age groups. This was done by calculating the age distribution of HAT, malaria and tuberculosis cases recorded at the Serere health centre and comparing it qualitatively to the age-productivity function curve [2].

A final comparison assessed the relative hospital costs of HAT to all other diseases for which patients were admitted at the Serere health centre. For each disease, total hospital stay (for 1999 non-HAT diseases) was calculated and multiplied by the standard cost per hospital day as described above. Tuberculosis was excluded because of the current strategy of direct observed therapy (DOTS) in lieu of hospital admission. HIV/AIDS was also excluded because of its association with concurrent opportunistic infections.

## Results

The data at Serere health centre included 72 cases (4 reported deaths) of HAT in 1999, 51 cases (3 reported deaths) in 2000, 45 cases (2 reported deaths) in 2001, 87 cases (1 reported death) in 2002, 121 cases (8 reported deaths) in 2003, 99 cases (5 reported deaths) in 2004 and 93 cases (4 reported deaths) in 2005. The 1999 records included 11,228 diagnosed malaria cases (including 763 cases of inpatient severe malaria), with 104 cases in the tuberculosis register.

### Disability Adjusted Life Year (DALY) estimates for Rhodesiense HAT

The modelled annual age-specific number of reported cases, reported deaths, estimated unreported cases/deaths (assuming a 0 or 69% under-reporting rate), and non-age-weighted discounted YLL (DYLL) are listed in Table 2. If we consider that all cases were reported (0% under-reporting) then the non-age weighted total YLL would be only 65. Of greater importance was under-reporting; only 4 (95%CI = 1 – 8; median = 4) deaths were recorded at the health centre while we estimate that an additional 59 (95% CI = 25 – 109; median = 57) deaths due to HAT went unreported [30]. The age-specific, non-age-weighted, total YLLs for a 69% under-reporting rate are also listed, summing to a total of 1136 (95% CI = 493 – 2132; median = 1080). If age-weighting is applied, the total YLL estimate is 1405 (95% CI = 609 – 2657; median = 1333).

**Table 2:** Years of Life Lost (YLL) due to rhodesiense sleeping sickness in Serere, Uganda. Values are mean values (with lower and upper 95% confidence intervals) from 10,000 Monte-Carlo simulations, derived from data from 1999–2005.

Age of onset	Study population	Annual reported non-fatal early cases	Annual reported non-fatal late cases	Annual reported deaths	Annual YLLs with 0% under-reporting (age weighted)	Annual YLLs with 0% under-reporting (non-age weighted)	Annual deaths with 69% under-reporting	Annual YLLs with 69% under-reporting (age weighted)	Annual YLLs with 69% under-reporting (non-age weighted)
0–4	7145	1.6 (0–3)	2.1 (0–4)	0.1 (0–1)	4 (0–28)	3.5 (0–24.8)	2.8 (0.5–5.5)	86.9 (17–170)	71.4 (13.9–140.1)
5–14	12432	5.7 (2–13)	11.4 (3–17)	1 (0–3)	33.2 (0–100.5)	24 (0–73.3)	12.7 (4.2–27)	423.2 (141.1–902.5)	308.7 (102.4–660.5)
15–29	12687	9.9 (3–16)	16.7 (9–23)	0.7 (0–2)	19.7 (0–52.8)	14.9 (0–40.4)	19 (7–34.8)	552.7 (199.1–1027.2)	406.3 (147.3–752.8)
30–44	6857	4.6 (1–8)	9.4 (4–18)	0.6 (0–1)	10.3 (0–18.7)	9.8 (0–18)	10.1 (4.4–21.8)	210.6 (90.6–444.7)	184.6 (79.8–391.8)
45–59	4668	2.4 (0–5)	6.7 (3–10)	0.1 (0–1)	1.9 (0–13.1)	2.1 (0–15)	6.5 (2.1–12.2)	83.4 (28.6–154.3)	93.3 (31.4–171.5)
60–69	1850	2.4 (0–5)	4.1 (3–7)	0.9 (0–2)	6.1 (0–16.4)	8.7 (0–22.6)	5.4 (1.6–10.4)	39.4 (10.4–74.3)	55.6 (15–105.9)
70–79	781	0.1 (0–1)	2.3 (0–4)	0.3 (0–1)	1.1 (0–4.4)	1.9 (0–7.5)	1.9 (0–4.6)	8 (0–19.2)	13.5 (0–32.5)
80+	235	0.6 (0–3)	0.4 (0–2)	0.1 (0–1)	0.2 (0–1.2)	0.4 (0–3)	0.8 (0–3.5)	1.6 (0–6.5)	3.5 (0–14.3)

Totals	**46655**	**27.3 (7–48)**	**53.3 (24–73)**	**3.9 (1–8)**	**76.4 (17.3–160)**	**65.4 (16.2–132.1)**	**59.2 (25.2–109.1)**	**1405.8 (608.8–2657.2)**	**1136.8 (493–2132.2)**

The years of life lived with disability (YLD), for cases that recovered due to treatment are listed in Table 3. The non-weighted total YLD is 20.5 (95% CI = 9.2 – 30.0; median = 21.9). If age-weighting is applied the total YLD estimate increases marginally to 24.7 (95% CI = 11.4 – 37.1; median = 25.7).

**Table 3:** Years of Life Lived with Disability (YLD) due to rhodesiense sleeping sickness in Serere, Uganda. Disability weights of 0.21 and 0.81 were used for early and late stage cases, respectively. Values are mean values (with 95% confidence intervals) from 10,000 Monte-Carlo simulations, derived from data from 1999–2005.

Age of onset (years)	Number of recovered patients (early+late)	Total non-age weighted YLD (early+late)	Total age weighted YLD (early+late)
0–4	3.7 (1–6)	0.9 (0–1.5)	0.3 (0–0.6)
5–14	17.1 (6–29)	4.4 (1.2–6.9)	4.9 (1.3–7.7)
15–29	26.6 (13–38)	6.5 (3.3–9.4)	9.9 (5–14.3)
30–44	14 (9–25)	3.6 (1.8–7.1)	5 (2.4–9.9)
45–59	9.1 (4–14)	2.5 (1.1–3.9)	2.7 (1.1–4.2)
60–69	6.6 (3–10)	1.6 (1–2.7)	1.3 (0.8–2.2)
70–79	2.4 (0–4)	0.8 (0–1.5)	0.5 (0–0.9)
80+	1 (0–3)	0.2 (0–0.8)	0.1 (0–0.4)
			
**Totals**	**80.6 (45–121)**	**20.5 (9.2–30)**	**24.7 (11.4–37.1)**

The total estimated DALYs for rhodesiense HAT are shown in Table 4. Based on 69% under-reporting and no age-weighting, the mean annual DALY burden in Serere was 1157 (95% CI = 504 – 2159; median = 1103) compared to 86 (95% CI = 35 – 162; median = 82) based on 0% under reporting (Table 4); the majority (93%) of this DALY burden was due to mortality in unreported cases. When applying a disability weight per late stage HAT episode of 0.35 [18], the estimated DALY burden for this population is 1148 (95% CI = 499 – 2146; median = 1092), reflecting again the emphasis on mortality as the main driver in overall burden in this study.

**Table 4:** Disability-Adjusted Life Years due to rhodesiense sleeping sickness in Serere, Uganda. Values are mean values (with 95% confidence intervals) from 10,000 Monte-Carlo simulations, derived from data from 1999–2005. The DALY score for each age group = YLL + YLD for that age group.

	DALYs with age weighting		DALYs with no age weighting	
Age group	Assuming 0% under-reporting	Assuming 69% under-reporting	Assuming 0% under-reporting	Assuming 69% under-reporting
0–4	4.3 (0–28.6)	87.3 (17–170.6)	4.4 (0–26.3)	72.2 (13.9–141.6)
5–14	38.2 (4.5–108.1)	428.1 (142.4–909.5)	28.4 (4–80)	313.1 (103.6–666.7)
15–29	29.6 (5–65.5)	562.6 (204.6–1040.8)	21.4 (3.3–48.8)	412.8 (151–761.2)
30–44	15.3 (2.4–27.2)	215.6 (94.1–453.8)	13.4 (1.8–23.4)	188.2 (82.2–398.5)
45–59	4.6 (1.1–16.3)	86 (29.8–158.2)	4.7 (1.1–18)	95.9 (32.5–175.2)
60–69	7.4 (0.8–17.5)	40.7 (11.2–75.9)	10.3 (1–24)	57.2 (16.1–108)
70–79	1.6 (0–5.4)	8.5 (0–20.1)	2.7 (0–9)	14.3 (0–33.9)
80+	0.3 (0–1.2)	1.7 (0–6.7)	0.6 (0–3.1)	3.7 (0–14.6)
				
**Total**	**101.2 (39.8–197.1)**	**1430.5 (623.9–2691.2)**	**85.9 (35.1–162.1)**	**1157.3 (504.3–2158.7)**

### Costs to the local health system of rhodesiense HAT

Between 1999 and 2005, the mean number of recorded HAT patients per year in Serere was 84.5 (95% CI = 47 – 129; median = 88); with a mean hospital stay per patient of 47 (95% CI = 37 – 58; median = 46) days. The standard daily health cost estimated by staff at the Serere health centre was US$ 1.75–2.35. The mean total annual cost of HAT to the Serere health system was US$ 7649 (95% CI = 3668 – 12,773; median = 7744). Drug costs are not met by the district and national health services as drugs are donated by the World Health Organization following agreements with the manufacturers [40]. The mean annual costs of these drugs for late stage cases (melarsoprol) was US$ 3357 (95% CI = 1512 – 4599; median = 3780) and that for early stage cases (suramin) US$ 955 (95% CI = 245 – 1680; median = 1085), giving a total of an additional US$ 4312 (95% CI = 2247 – 6279; median = 4445) for drugs per year; therefore, the total costs (drugs+hospital costs) of treating HAT in Serere was US$ 11,961 (95% CI = 5950 – 19,025; median = 12,362), or US$ 147 (95% CI = 125 – 173; median = 147) per patient.

We modelled the DALYs averted as a result of the hospital intervention by comparing annual mortality i) with the hospital treatment (mean of 4 annual deaths as modelled above) and ii) under the alternate assumption that all cases presenting at the hospital die, as would be the case in the absence of treatment. This part of the analysis did not account for under-reporting in the community, and we used non age-weighted outputs. If each patient presenting to hospital died, this would represent 1570 (95% CI = 876 – 2401; median = 1710) DALYs lost, while we have already shown that 86 (95% CI = 35 – 162) DALYs were lost with the observed intervention. The hospital-based intervention, as observed, therefore saved 1484 (95% CI = 841 – 2239; median = 1488) DALYs, at a cost to the health authorities of US$ 11,961 (95% CI = 5950 – 19,025), including drug costs – see above. This equates to US$ 8.06 (95% CI = US$ 7.1 – 8.5; median = 8.31) per DALY averted for the reported, hospitalised cases.

### Comparison to local burdens of other diseases

The most common disease at the Serere health centre was malaria. A total of 11,228 cases were diagnosed in 1999 (see Table 5), mainly by using clinical signs, with only a few cases being confirmed parasitologically. All recorded HAT cases were parasitologically confirmed. The relative burden of malaria and HAT differs greatly depending on whether DALY estimates or total case numbers are compared (see Table 5). This is because of the relatively lower reported case-fatality rate for malaria (2 out of 11,228 compared to a mean of 4 out of a mean of 80 reported cases for HAT) and the shorter duration of illness for a malaria patient (average of 4 days), with most being treated as outpatients; all diagnosed HAT cases were hospitalised as inpatients. DALYs, particularly the YLL component, depend on an accurate estimation of reported deaths. If we assume no under- or over- reporting of malaria [41], the mean DALY estimate for malaria in Serere is 271 and that for HAT 86 (Table 5). 82% (251) of the DALY burden for malaria was accounted for by children less than 5 years, a finding consistent with other studies [42,43].

**Table 5:** Comparison of the mean burden due to sleeping sickness and malaria for Serere health facility, Soroti District, Uganda (data for malaria are from 1999 records). The YLLs, YLDs and DALYs figures in parenthesises where calculated with age weighting, while those outside the parentheses were calculated without age-weighting.

	Sleeping sickness			Ratio of sleeping sickness : malaria	
	No under reporting	69% under-reporting	Malaria Observed	No under-reporting	69% under-reporting
Cases	84.5	143.5	11,228	**1 : 133**	**1 : 78**
In-patients	84.5	84.5	763	1 : 9.0	1 : 9.0
Deaths	4	59	2	1 : 0.50	1 : 0.03
YLLs	65 (76)	1137 (1406)	150.4 (176.6)	1:2.3 (1 : 2.3)	1 : 0.1 (1 : 0.1)
YLDs	21 (25)	21 (25)	120.3 (130.4)	1 : 5.7 (1 : 5.2)	1 : 5.7 (1 : 5.2)
DALYs	86 (101)	1157 (1430)	270.7 (306.9)	**1 : 3.1 (1 : 3.0)**	**1 : 0.23 (1 : 0.21)**

**Figure 1:**
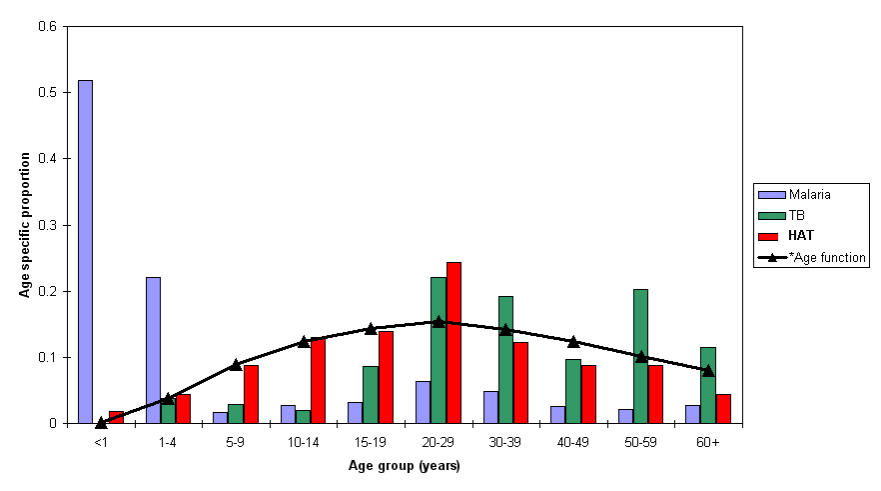
Age distribution of malaria, sleeping sickness and tuberculosis patients admitted to Serere health centre compared to the age- productivity function curve [2].

The age distribution of HAT, malaria and tuberculosis cases at the Serere health centre differed. Figure 1 shows the age distribution for 763 severe malaria cases admitted to the Serere health centre in 1999, 104 tuberculosis cases treated in 1999 and 115 HAT cases admitted to the Serere health centre between January 1999 and August 2000. The age pattern of HAT cases has a peak in the 20–29 age category. HAT affects the most productive age groups as illustrated by the close association between its age distribution and the age-productivity function curve of Murray [2] (see Figure 1). For malaria, the age distribution is skewed with the majority of cases being less than 5 years of age. The tuberculosis age distribution is skewed with the majority of cases occurring among adults.

**Figure 2:**
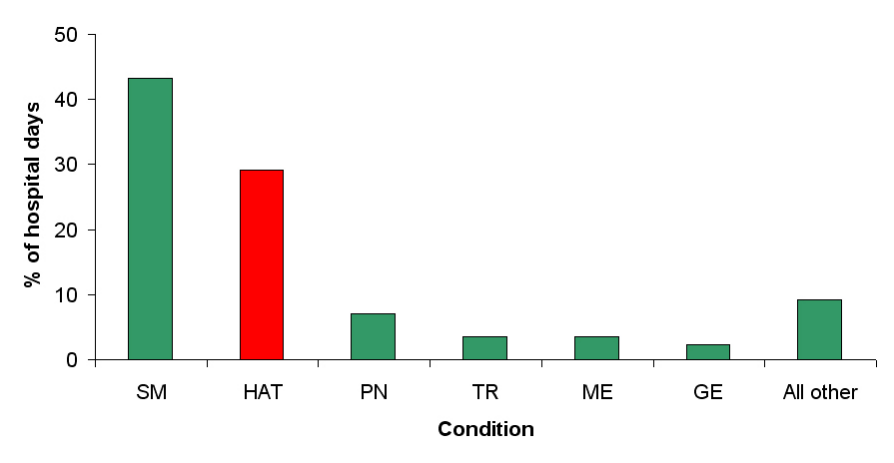
Percent of hospital days for inpatients by condition at Serere health centre in 1999.

The proportion of hospital days by disease provides a reasonable estimate of the relative costs to the Serere health system of different diseases, as the daily hospital costs are relatively constant across diseases. The proportions of hospital days for the 6 most common causes of admission are shown in Figure 2. In 1999, severe malaria accounted for approximately 40% of hospital days and HAT, 30%.

## Discussion

Many countries in sub-Saharan Africa (SSA) are devolving decision-making for health service delivery to the local level. Local burden of disease estimates can play an important role in health sub-districts rationally allocating resources to the highest priority diseases [22]. The DALY is currently the most widely accepted measure for estimating the burden of human disease; in this study, we have shown that with relatively small-scale datasets available from a local in-patient health centre, useful estimates of relative burden can be made. Thus, DALY estimates for local planning purposes and the relative ranking of adverse health conditions can be readily estimated from local health centre data using the data outlined in Table 1.

WHO estimates the treatment costs (hospitalisation and drugs) per patient of HAT to be in the order of greater than US $120 [9]; WHO estimates are of the same order as our estimates of US$147. In addition to the DALY estimates we present, we show that hospital costs, which are also an important planning tool for local health decision makers, can be simply derived from in-patient records, particularly in countries with good reporting systems [44]. For convenience and ease of comparison, we have assumed that hospitalisation costs are relatively standard across diseases; however, drugs for the treatment of HAT are administered parentally and, due to toxicity, complications are frequent, increasing the costs of treatment; our estimates are thus conservative. In addition, we have not accounted for costs beyond those to the local health system, such as costs to individual patients. These may vary by disease, particularly if appropriate diagnostic facilities are lacking in rural areas [39].

This is the first study to estimate the DALY specifically for *rhodesiense *as opposed to *gambiense *HAT; local estimates for *gambiense *HAT are available elsewhere [17]. Given that HAT of both types is invariably fatal if not treated and that HAT surveillance is relatively weak throughout sub-Saharan Africa [9], it is not surprising that mortality, particularly mortality due to unreported cases, dominates the DALY estimate; this is a reflection of the neglected status of this disease. Given that 95% of treatments for rhodesiense HAT were successful at Serere health centre, resulting in a cost per DALY averted (compared to a "do-nothing" scenario) of under US$ 10 without accounting for under-reporting, there are great potential benefits in enhancing strategies for identifying unreported HAT cases in the community, and improving reporting rates. As a general rule, an attractive intervention in developing countries is one in which the cost per DALY averted is under US$ 150, and a highly attractive intervention is one with a cost per DALY of under US$ 25 [45]. Resources targeted at improving reporting and reducing the hidden burden of HAT would therefore be highly cost-effective. These results also serve to further emphasise the need for enhanced surveillance for neglected zoonotic diseases more generally.

Our estimates of disease burden relate to a period when the catchment area of the hospital was experiencing an outbreak of *T.b. rhodesiense *HAT [21], the disease was previously absent but has become well-established in this north-eastern region. While the incidence of reported HAT appears to have decreased across Africa generally in the past 5 years [46], Uganda has seen the expansion of the *T.b. rhodesiense*-affected zone and the potential for a much larger number of cases. At regional and local levels, information about the disease burden over this time period could be a valuable addition to the decision making process for planning public health interventions [24].

Because of its high mortality, relatively acute and severe clinical course and age distribution, rhodesiense HAT had a DALY estimate that was high compared to malaria. The main difficulty in comparing HAT and malaria DALYs is the problem of estimating the relative rate of under-reporting of the two diseases based on hospital data. Though there were 2 reported malaria deaths there are likely to have been more deaths occurring in the community, which were not logged by the health system. The number of reported cases of malaria (11,228) is 133× higher than the estimated mean of 85 reported HAT cases per year; these numbers may not be a good reflection of the relative incidence of the two diseases, as the standards for diagnosis differed. For example, many fevers due to other causes (including early stage HAT) may be diagnosed as malaria in hyper-endemic areas resulting in over-reporting. Amexo [41] reported that in Uganda, clinical diagnosis of malaria without parasitological confirmation could over-estimate malaria incidence by 43–57%. Conversely, many patients, even with severe malaria, will self-treat and not report to the health units. The magnitude of mis-reporting and under-reporting for a range of diseases in Serere deserves further attention. HAT is always confirmed through parasitological means, giving us confidence that the HAT incidence, is, at least, not over-reported. Another complication in comparing the relative burdens of malaria and HAT is their differing patterns of occurrence, with malaria having a more widespread incidence than HAT. However, our study does accurately present the relative burden of the two diseases in that sub-set of the population that reports for treatment at the health centre.

Our study did not account for any post-treatment disability resulting from HAT infection. This is difficult to quantify; studies in Cameroon [38,47] have shown that children with a past history of *T.b. gambiense *HAT had significantly lower weight, height and mid-arm circumference and spent more years in school. Long term disability was particularly noted in patients treated for late-stage disease. No similar studies exist for *T.b. rhodesiense*.

Recent economic impact assessments of trypanosomiasis control in livestock have excluded the public health burden of the disease [10,11,48]. Our *T. b. rhodesiense *HAT DALY estimates in eastern Uganda indicate that where *T. b. rhodesiense *HAT outbreaks occur, the disease has an important impact on human health. This public health impact needs to be considered in overall tsetse and trypanosomiasis control planning. Trypanosomiasis in cattle is an important veterinary problem in much of Uganda and East Africa, and both tsetse control and treatment of cattle fall under the mandate of the Ministries of Agriculture of most governments in Africa. The treatment of livestock for trypanosomiasis is one of the important control strategies where *T. b. rhodesiense *HAT occurs because cattle are the main reservoir of zoonotic human-infective trypanosomes [12-14]. This study highlights some preliminary analyses [49] that have shown that coordinated control of animal and human trypanosomiases would be economically beneficial, with benefits to both the livestock and public health sectors by reducing the burden on health services; further work is required to estimate the DALY reduction from a given effort in controlling trypanosomiasis in the animal reservoir.

Our DALY estimate for rhodesiense HAT in Serere, Uganda, has shown that mortality due to unreported cases was the major contributor to its burden on the local population, while reported cases of *T.b. rhodesiense *HAT alone consumed 30% of in-patient time in Serere health centre; the disease has now become well established in the region [21], and continues to spread to areas where under-reporting is likely to be accentuated [50]. The parameters used in our model could be usefully applied to other settings in East Africa. Thirteen countries are considered to be endemic for *T.b. rhodesiense *[46], with varying incidence and under-reporting rates; efforts need to be mustered to make best use of what data are available in treatment centres and hospitals in each HAT focus, to estimate the level of under-reporting in a manner appropriate to each focus, and to use this to estimate DALYs lost due to the disease.

Finally, our analysis employs a stochastic framework to estimate both the disease and economic burden of HAT in this community. Early disease burden assessments were deterministic in nature [3,16], where uncertainty in the diverse inputs for the calculations is not reflected in the outputs. This has been the case even for large-scale national and regional studies [26,51], although there are increasing numbers of studies on neglected diseases that do quantify uncertainty [8,52-54]. Quantifying uncertainty explicitly increases the value of DALY outputs as a tool in decision making, and, importantly, helps to identify gaps in knowledge for some parameters.

## Conclusion

The relative burden of HAT can be usefully assessed using a variety of methods, especially the DALY, at regional and sub-regional levels using data from local hospital records and reporting systems. This is particularly important for focal diseases which may greatly affect one community and be absent from another. The disability weighting used in previous DALY assessments of HAT did not appropriately account for the different YLDs for early and late stage disease; in addition, as we have emphasised here, *T.b. rhodesiense *and *T.b. gambiense *should be separately accounted for in wider scale burden assessments in future because they present as such clinically distinct diseases. In our study site centred on one treatment centre, *T.b. rhodesiense *HAT resulted in a mean of 1157 DALYs lost per year (without age-weighting), with unreported cases representing the greatest majority of this burden.

## Competing interests

The author(s) declare that they have no competing interests.

## Authors' contributions

All authors conceived of the study. EMF, MO and PGC collected and analysed the data. All authors were involved in drafting the manuscript and all have read and approved the final manuscript.

## Pre-publication history

The pre-publication history for this paper can be accessed here:


